# The relationship between coronary stenosis morphology and fractional flow reserve: a computational fluid dynamics modelling study

**DOI:** 10.1093/ehjdh/ztab075

**Published:** 2021-08-15

**Authors:** Roberto T F Newcombe, Rebecca C Gosling, Vignesh Rammohan, Patricia V Lawford, D Rodney Hose, Julian P Gunn, Paul D Morris

**Affiliations:** 1 Department of Infection, Immunity and Cardiovascular Disease, The Medical School, The University of Sheffield, Beech Hill Road, Sheffield S102RX, UK; 2 Insigneo Institute for in Silico Medicine, Frederick Mappin Building, Mappin St, Sheffield S1 3JD, UK; 3 Department of Cardiology, Chesterman Building, Sheffield Teaching Hospitals NHS Foundation Trust, Herries Road, Sheffield S5 7AU, UK

**Keywords:** Coronary artery disease, Fractional flow reserve, Computer modelling

## Abstract

**Aims:**

International guidelines mandate the use of fractional flow reserve (FFR) and/or non-hyperaemic pressure ratios to assess the physiological significance of moderate coronary artery lesions to guide revascularization decisions. However, they remain underused such that visual estimation of lesion severity continues to be the predominant decision-making tool. It would be pragmatic to have an improved understanding of the relationship between lesion morphology and haemodynamics. The aim of this study was to compute virtual FFR (vFFR) in idealized coronary artery geometries with a variety of stenosis and vessel characteristics.

**Methods and results:**

Coronary artery geometries were modelled, based upon physiologically realistic branched arteries. Common stenosis characteristics were studied, including % narrowing, length, eccentricity, shape, number, position relative to branch, and distal (myocardial) resistance. Computational fluid dynamics modelling was used to calculate vFFRs using the VIRTUheart™ system. Percentage lesion severity had the greatest effect upon FFR. Any ≥80% diameter stenosis in two views (i.e. concentric) was physiologically significant (FFR ≤ 0.80), irrespective of length, shape, or vessel diameter. Almost all eccentric stenoses and all 50% concentric stenoses were physiologically non-significant, whilst 70% uniform concentric stenoses about 10 mm long straddled the ischaemic threshold (FFR 0.80). A low microvascular resistance (MVR) reduced FFR on average by 0.05, and a high MVR increased it by 0.03.

**Conclusion:**

Using computational modelling, we have produced an analysis of vFFR that relates stenosis characteristics to haemodynamic significance. The strongest predictor of a positive vFFR was a concentric, ≥80% diameter stenosis. The importance of MVR was quantified. Other lesion characteristics have a limited impact.

## Graphical Abstract

**Figure:**
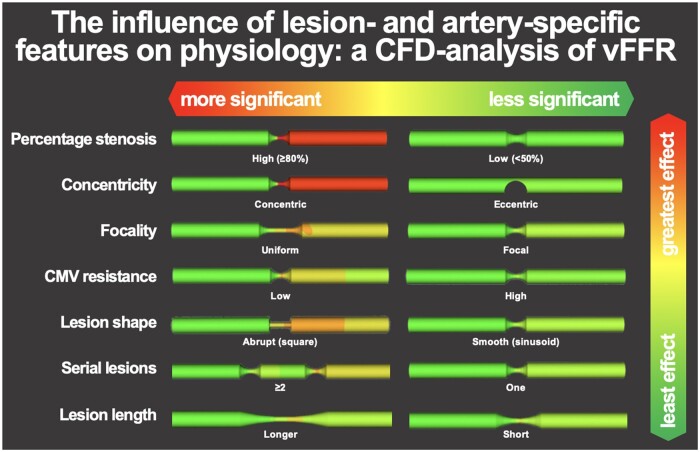

## Introduction

Invasive coronary angiography remains the most commonly used tool to assess patients and guide coronary revascularization. Visually assessing the impact of a stenosis upon blood flow however, is difficult.^1^^,^^2^ Fractional flow reserve (FFR) and related physiological indices can accurately identify ischaemia-inducing stenoses,^3^^,^^4^ and can improve outcomes when treatment is based upon selecting lesions with FFR ≤0.80 compared with angiography alone.^5^^,^^6^ FFR is, however, under-used.^7^^,^^8^ ‘Virtual’ FFR (vFFR) is computed from medical images, such as computed tomography or, in this case, the invasive angiogram, by reconstructing a series of coronary artery images taken from orthogonal planes in virtual space into a 3D geometry, which the rules of computational fluid dynamics (CFD) can be applied to in order calculate blood flow.^9^^,^^10^ In a clinical context models of vFFR predict physiological lesion significance with a sensitivity of 89% and specificity of 90% as reported in a large meta-analysis of vFFR which included the VIRTUheart model of coronary physiology used in this study.^11^ This technology has been used to predict the physiological response to percutaneous coronary intervention and can be readily adapted to construct any number or type of idealized stenoses and their corresponding vFFRs.^12^^,^^13^

Our aim was to analyse vFFR in commonly encountered patterns of coronary artery disease and anatomy, to provide improved understanding of coronary anatomy and physiology when viewing an angiogram.

## Methods

### Study design and setting

This study was carried out using simple, idealized, 3D tubular geometries, based upon patterns of disease and physiological measurements informed by clinical data encountered in real-world practice. The study was performed *in silico* at the University of Sheffield.

### Geometries

Tubular geometries were generated using ANSYS 18.2 DesignModeler™. We were able to define the characteristics of each stenosis and vessel generated to 100% accuracy, without any ambiguity, thereby removing any degree of inter-observer variability. They contained stenoses of various shapes, severity, number and length, within a basic vessel template, comprising a main vessel which was a rigid, straight tube, 50–100 mm long, 3.5 mm in diameter. Selected important clinical variants of the basic model were constructed and analysed. To maximize clinical relevance and limit the number of permutations, we confined our models to those that would produce vFFRs which straddled the 0.80 treatment threshold. Therefore, no diameter stenoses (DS) >90% and <50% were studied, because early experimentation with permutations of these stenoses revealed they all have FFR <0.70 and >0.80, respectively (see Supplementary material online, *Table S1*).

We studied a total of 174 simulations incorporating 130 geometries. These were all variants of a basic pattern of a rigid 3.5 mm diameter tube of up to 100 mm length containing one or more stenoses with the characteristics defined below. Larger and smaller parent vessels were not studied, on the assumption that the physiology would be the same, if the anatomical proportions of the model were the same.

### Cross-sectional shape

We studied 50%, 70%, and 80% DS, in both concentric (narrowed in both orthogonal planes) and eccentric (narrowed in one plane) configurations (*Table 1*).

**Table 1:** Definitions of % DS and their corresponding CSAs

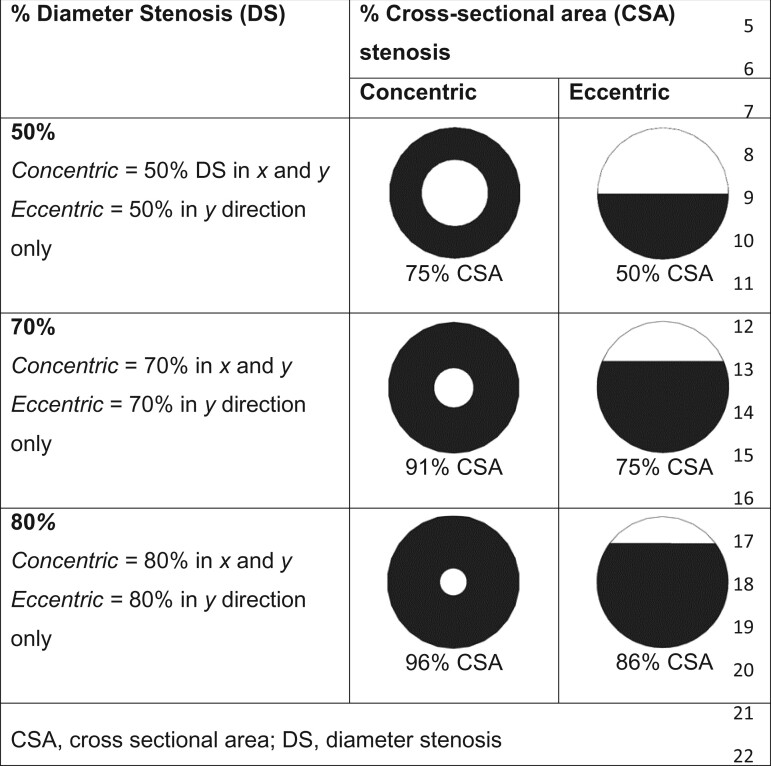

CSA, cross-sectional area; DS, diameter stenosis.

### Lesion length

**Figure 1:**
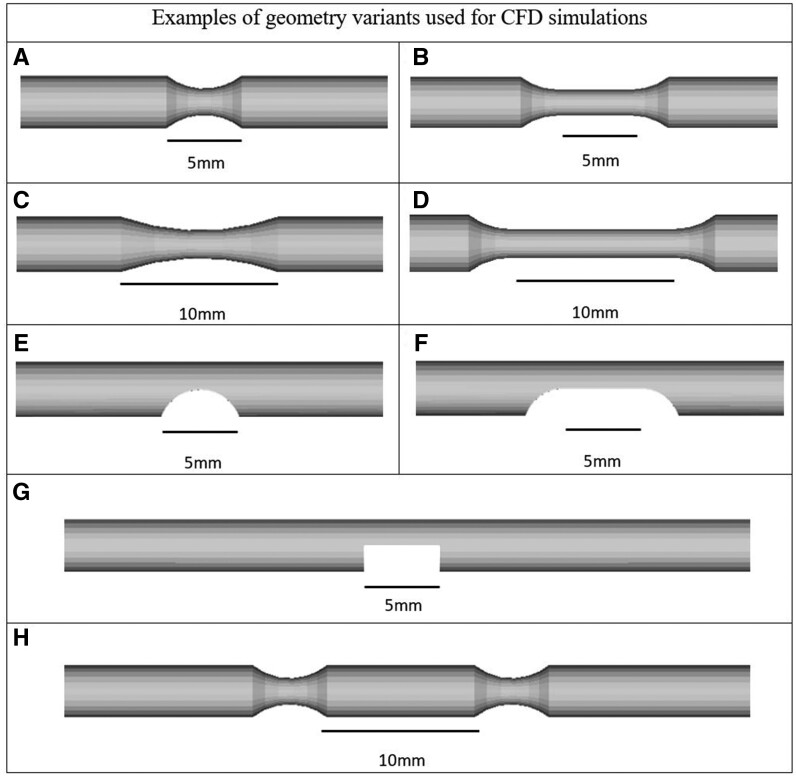
Examples of geometry variants used for simulations. Examples of variations upon the standard artery geometry viewed longitudinally. All % stenoses quoted are diameter stenoses (DS). (*A*) A 50%, concentric, rounded, 5 mm long focal stenosis; (*B*) a 50%, concentric, rounded, 5 mm long uniform stenosis; (*C*) a 50%, concentric, rounded, 10 mm long focal stenosis; (*D*) a 50%, concentric, rounded, 10 mm long uniform stenosis; (*E*) a 50%, eccentric, rounded 5 mm focal stenosis (*F*) a 50% eccentric, rounded, 5 mm long uniform stenosis; (*G*) a 50%, eccentric, rectangular longitudinal section 5 mm long stenosis; (*H*) two serial, concentric, rounded, 5 mm long focal, 50% stenoses.

The basic stenosis was 5 mm long with a rounded profile in longitudinal projection. Lesions were also created in lengths of 10, 20, and 30 mm, by either inserting straight segments (‘uniform’ stenosis, *Figure 1D*), or using a curve which reached maximum DS at mid-point (‘focal’ stenosis, *Figure 1C*).

### Longitudinal lesion morphology

We studied 5 mm stenoses that were rectangular in longitudinal section (*Figure 1G*) as well ones which were rounded, whilst maintaining the same spread of vessel sizes, eccentricity or concentricity, and % stenosis. For these lesions, we also studied ‘uniform’ or ‘focal’ stenoses (see above; *Figure 1A and B*).

### Serial stenoses

We studied two and three serial stenoses (*Figure 1H*), 10 mm apart, whilst maintaining the same spread of characteristics of vessel sizes, eccentricity or concentricity, uniformity and % stenosis.

### Branches

We studied branched geometries at 45°, with combinations of commonly encountered diameters of the proximal main branch (PMB) and distal main branch (DMB). The side branch (SB) diameter was then calculated using each of three commonly accepted bifurcation laws (Murray’s, Finet’s, and Huo-Kassab) that outline the relationship between the diameters of the main and daughter branches.^14–16^ This enabled comparison between otherwise equivalent geometries to study the effect of the three laws on the vFFR. The CFD simulation assumed continuity of static pressure and flow at the bifurcation points but, because of the discontinuity at the branch point, local flow disturbances, and momentum effects may have been underrepresented in more tangential branches. However, these effects are second order and this has previously been shown to reproduce accurate vFFR results and better characterize bifurcation haemodynamics relative to 1D and 2D model representations (unpublished data).^17^

### Virtual fractional flow reserve

Geometries were processed using the Sheffield VIRTUheart™ workflow, which incorporated segmentation, mesh formation, CFD simulation using ANSYS CFX™, and portrayal of vFFR in colour, from green (FFR 1.00, no flow limitation), through yellow/orange (FFR 0.80, borderline) to red (FFR 0.60, severe flow limitation). The FFR value quoted was at the outlet of each model. A ‘lower’ or ‘reduced’ vFFR was taken throughout to refer to a lower numerical value (e.g., 0.76 is ‘lower’ than 0.86). A ‘positive’ FFR refers to a physiologically significant FFR value equal to, or below the ischaemic threshold (≤0.80).

### Microvascular resistance

We used a standard value of microvascular resistance (MVR) as the distal boundary condition. This was a generic average (8.721E + 9 Pa.s.m^−3^) obtained from a previously studied patient cohort.^9^ However, because MVR is variable between individuals, we decided to select geometries containing 70% DS to vary MVR by ± 2E9 Pa.s.m^−3^. This represents a variation in MVR of 20–25% (23%) which is representative of values we had from the aforementioned patient cohort, as it aligned approximately with the upper and lower quartiles and was thought to be of sufficient magnitude to capture the impact of MVR on FFR in our geometries. MVR (and flow) were assumed to vary proportionately with the diameter of the vessel, so results were given for 3.5 mm diameter vessels only.

## Results

### Effect of lesion severity, eccentricity, and shape

For a single, short (5 mm long), rounded, focal, 70%, concentric stenosis, the vFFR was 0.90; and for an equivalent 80% concentric stenosis it was 0.68 (*Table 2*). No eccentric stenoses up to, and including, 80% produced a positive vFFR. All concentric lesions with an abrupt (‘rectangular’) profile produced lower vFFR values than their rounded equivalents. This effect became more marked with lesion severity; the 70% concentric rectangular lesion producing a vFFR 0.80, and the equivalent 80% lesion 0.50. A rectangular shape had a negligible effect upon the vFFRs of eccentric stenoses.

**Table 2:** Effect of lesion severity, eccentricity, and shape upon vFFR

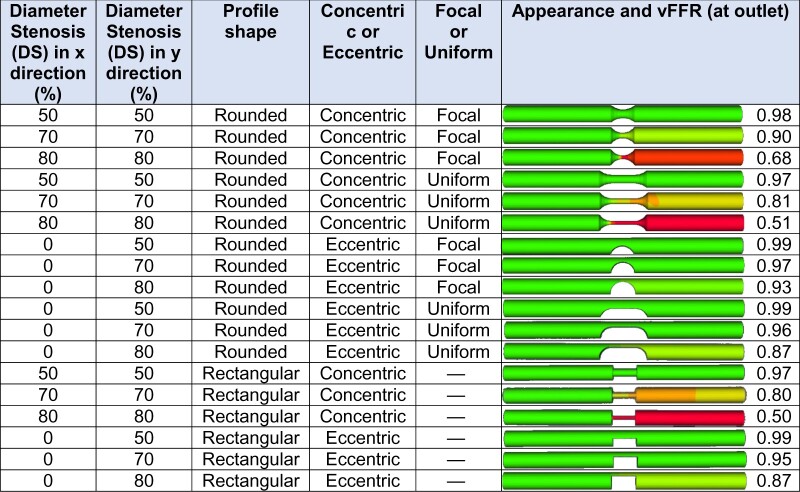

DS, diameter stenosis; vFFR, virtual fractional flow reserve.

For a single, short (5 mm) lesion, this table displays the effects of diameter stenosis (DS) concentricity (narrowed in *X and Y*) or eccentricity (narrowed in *Y* only), uniformity and shape (rounded or rectangular) upon vFFR. The vessels are 3.5 mm diameter, 50 mm long, and the microvascular resistance is set to 8.721E + 9 Pa.s.m^−3^.

### Effect of lesion length

For a single, 10 mm long, rounded, 70%, concentric, focal stenosis, the vFFR was 0.89, and with a uniform (rather than focal) narrowing, the vFFR was 0.77 (*Table 3*). For the focal shape, 20 and 30 mm long 70% DS stenoses remained in the physiologically non-significant range, whereas the uniform stenoses were also positive at 20 and 30 mm. All 80% concentric stenoses produced positive vFFRs. The only significant eccentric lesions were those that were 20 and 30 mm long, uniform and had an 80% DS (0.75 and 0.69).

**Table 3:** Effect of lesion length upon vFFR

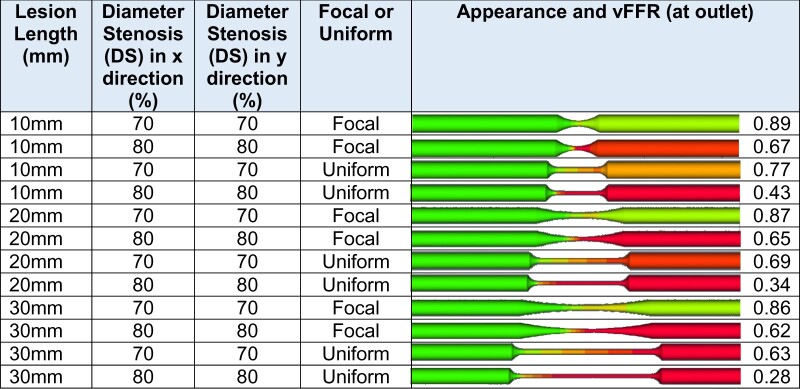

DS, diameter stenosis; vFFR, virtual fractional flow reserve.

For a single, long lesion, this table displays the effect of varying length, severity, and uniformity upon vFFR. All lesions shown are concentric. The vessels are 3.5 mm diameter and 100 mm long to accommodate the long lesions and to allow for flow stabilization, and the microvascular resistance is the standard previously quoted.

### Effect of serial lesions

For two, short (5 mm long), rounded, focal, 70%, concentric stenoses, the vFFR was 0.82; and for three stenoses of this pattern, it was 0.77 (*Table 4*). Two similar lesions, but of uniform narrowing, produced a vFFR 0.70, and three 0.63. All 80% concentric lesions produced a positive vFFR. The only 5 mm eccentric lesions producing a positive FFR were two and three serial uniform lesions, 80% DS (vFFR 0.78 and 0.70, respectively). On average, increasing lesion number from one to two had the greatest vFFR lowering effect. The addition of a third lesion had less of an effect on reducing vFFR (0.06 vs. 0.04).

**Table 4:** Effect of serial lesions upon vFFR

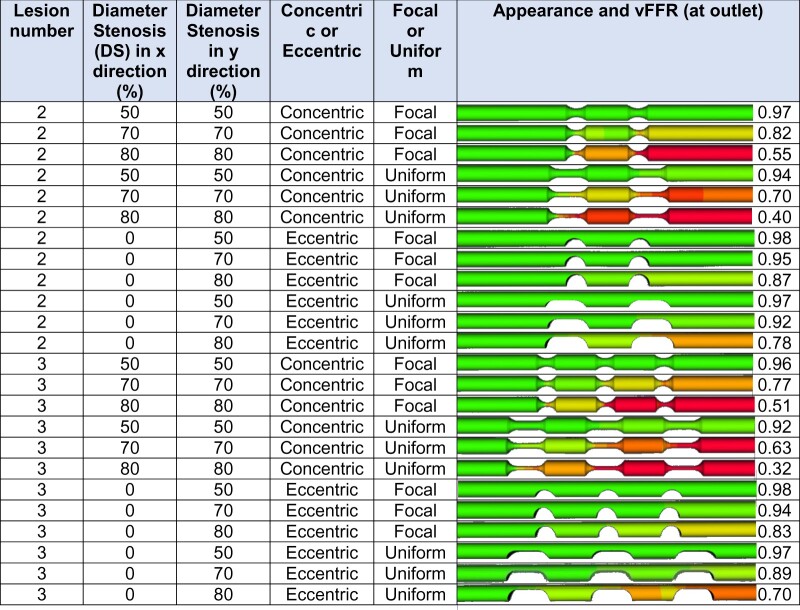

DS, diameter stenosis; vFFR, virtual fractional flow reserve.

For serial 5 mm stenoses, this table displays the effect of varying lesion number, severity, eccentricity, or concentricity and uniformity upon vFFR. The lesions are separated by 10 mm, vessels are 3.5 mm diameter and 100 mm long, and the microvascular resistance is the standard previously quoted.

### Effect of the microvascular resistance

For a single, short (5 mm long), rounded, focal, 70%, concentric stenosis, the vFFR with our standard value of resistance (8.721E + 9 Pa.s.m^−3^) was 0.90 (*Table 5*). Using our low MVR value (6.721E + 9 Pa.s.m^−3^) the vFFR was 0.85; and with our high MVR value (10.721E + 9 Pa.s.m^−3^) it was 0.92. For two serial 5 mm long focal stenoses, the equivalent high and low values were 0.76 and 0.86, respectively (the standard MVR generating a value of 0.82). In general, the low MVR reduced the vFFR by an average of 0.05, and the high MVR increased it by an average of 0.03.

**Table 5:** Effect of alterations to MVR upon vFFR

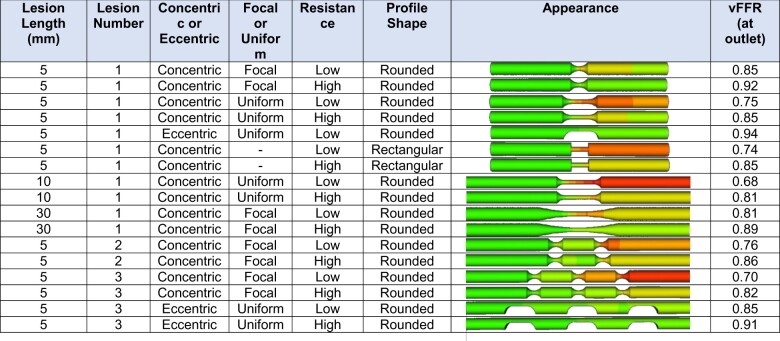

vFFR, virtual fractional flow reserve.

For vessels containing lesions of 70% DS, this table displays the effect upon vFFR of low (6.72E9 Pa.s.m^−3^) and high (10.72E9 Pa.s.m^−3^) distal (microvascular) resistance [the values in previous tables use the population average value (8.72E + 9Pa.s.m^−3^)]. To limit numbers displayed, we only include examples of each morphology that lie close to or either side of the 0.80 vFFR threshold. More severe (tighter, longer) lesions can be assumed to have a lower vFFR than those illustrated, and less severe, a higher vFFR.

### Effect of branches

For corresponding branched geometries containing different configurations of 5 mm rounded 70% concentric and eccentric focal stenoses, vFFR changed by at most 0.03 when varying the designated branch diameter law and the set DMB diameter (*Table 6*). None of the changes in vFFR resulted in a value <0.80 and there was no vFFR difference at all between corresponding geometries containing eccentric stenoses. Furthermore, the vFFR difference between comparable straight and branched geometries was also minimal. For example, the difference in vFFR between any straight vessel containing two ‘standard’ lesions and any branched geometry containing two identical ‘standard’ lesions in succession (one each in the PMB and SB, with the comparable vFFR recorded at SB outlet) was 0.02 at most.

**Table 6:** Effect of branches and diameter law upon vFFR

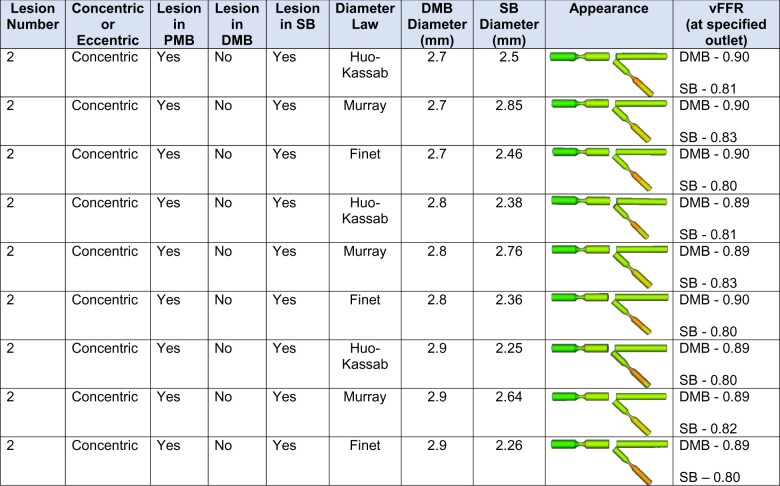

DMB, distal main branch; PMB, proximal main branch; SB, side branch; vFFR, virtual fractional flow reserve.

A selection of branched geometries containing 5 mm 70% focal concentric stenosis in the proximal main branch (PMB) and the side branch (SB). To limit numbers displayed, we only included corresponding examples where vFFR is changed in response to alterations in distal main branch (DMB) and SB diameter. PMB diameter was fixed at 3.5 mm and DMB diameter values were varied from 2.9 to 2.8 to 2.7. Huo-Kassab’s, Murray’s and Finet’s law were then used to calculate the diameter of the SB. The vFFR values are given at the outlet of the SB and DMB and thus factor in the effect of any lesion in the PMB.

## Discussion

### Summary

Using idealized vessel geometries and CFD simulation we have created a ‘library’ of virtual (computed) FFRs of commonly encountered patterns of coronary artery disease straddling the treatment threshold of ≤0.80. We found that no 50% lesions were significant. A uniform concentric 70% lesion 5 mm long has an FFR at about the ischaemic threshold. All concentric 80% lesions analyses were significant. Truly eccentric lesions are typically non-significant. The impact of other lesion characteristics, such as an abrupt outline and greater lesion length, are only important for borderline lesions. Serial lesions have a progressively smaller cumulative impact upon FFR. Microvascular resistance can have a large impact upon FFR. Lesions around (not at) a bifurcation have similar FFRs to equivalent lesions in unbranched vessels.

### Visual assessment

Lesions are often classified as mild, moderate or severe, but these terms lack precision. Whilst the human eye can identify a critically severe stenosis or a near normal artery, even expert assessments differ in between.^2^^,^^3^ There is a tendency for visual assessment to over-estimate more severe stenoses, and under-estimate mild ones.^18^ To try and resolve this ambiguity, clinicians quote lesion severity as percentage diameter stenosis. However, even this is subjective, and is also limited by a lack of specificity about whether the stenosis is seen in one or two planes. It is rare that a stenosis is truly entirely eccentric, but our work shows that an eccentric lesion, even if 80% narrowed in one view, is extremely unlikely to be physiologically significant, whereas if it is present in two orthogonal views, it is extremely likely. The quality of the angiogram, in terms of clarity, opacification and provision of truly orthogonal views, is also key. Nevertheless, even in study settings, the relationship between a visual assessment of the stenosis and measured FFR is poor; notably in the zone where decision-making is most important (i.e. about 50–90% DS).^19^ Quantitative angiography can be used to assist visual assessment but due to the difficulties of accurate edge detection is hardly an improvement^20^^,^^21^ and is not used in everyday practice.

### Importance of lesion shape, length, number, and branches

For a similar percent DS, an abrupt stepped ‘edge’ (idealized as rectangular here) is associated with a lower vFFR than a rounded one (see *Table 2* for a suitable example: a focal 70% concentric, rounded stenosis has FFR 0.90; whereas when there is an abrupt reduction in diameter and ‘squared off’ it is 0.80). This is because a sharper stenosis produces a tighter *vena contracta*, which is the minimum functional diameter that is smaller than the anatomical diameter of the stenosis itself, where velocity is at its maximum. Fluid must pass through this functional central flow region before once again readjusting to the anatomical boundary. Within a lesion, a uniform narrowing throughout gives rise to a lower FFR than a more focal stenosis (albeit highly simplified as a gentle curvature with the tightest segment at mid-lesion) (see *Table 3* for a suitable example: a 30 mm long, 70%, concentric, uniform stenosis has FFR 0.63; whereas when it is ‘focal’ (or gently tapered) it is 0.86). This is because the pressure losses due to viscous friction (Poiseuille effects) become amplified in a conduit with small radius. The presence of serial lesions does have an effect upon vFFR but this effect is incrementally less as each lesion is added (see *Table 4* for a suitable example: one concentric 5 mm focal 70% lesion has a vFFR of 0.90; two lesions in series produce a vFFR of 0.82 and three 0.77). This is because the definition of FFR is,

$$Q_{\text{stenosis}}/Q_{\text{normal}}$$

or alternatively

$$R_{\text{microvascular}}/\left(R_{\text{microvascular}}+R_{\text{stenosis}}\right)$$

This is non-linear, thus increasing the number of lesions, each of the same resistance, has incrementally less effect on FFR, even if the resistances themselves are linear. The presence of isolated lesions in branches (but not, in this paper, at the bifurcation point itself) can be regarded as individual or serial lesions (as described above) and a lesion in one daughter branch does not affect the FFR in another daughter branch (see *Table 6*). This phenomenon can be explained by the fact that presence of branches affects *absolute flow* but not the ratio of pressures across a lesion.^22^

### Importance of MVR

MVR is variable between patients and susceptible to factors such as microvascular disease, myocardial infarction, embolization and left ventricular hypertrophy.^23^^,^^24^ In a formal sensitivity analysis of vFFR, MVR contributed 70% of the variation of vFFR itself, i.e. it was the major determinant of vFFR, even above stenosis severity.^10^ If a direct, invasive measurement of distal pressure is known, the FFR (which measures the contribution of the epicardial lesion to flow limitation) is accurate, but in the absence of such a measurement, all models of coronary blood flow are susceptible to inaccuracy if the individual’s MVR deviates from the assumed or average level. Some models assume flow velocity, but this is also determined by MVR and thus susceptible to the same problems.^25^ This is of particular importance if the value of the vFFR of the lesion is close to the ischaemic threshold. A number of approaches to this problem have been taken, including trying to ‘tune’ the vFFR by using the vessel diameter (as used here in the experiment on branched geometries) or by using a flow parameter, such as the velocity of the contrast wave front.^26^ For the purposes of this work, such individual personalization was not necessary. More recent work has demonstrated that MVR can be quantified in absolute terms using similar CFD techniques, but this requires invasive measurement of distal pressure.^27^

### Limitations

First, the geometries modelled were relatively simple, consisting of a straight, rigid tube with regular idealized stenoses and were exaggerated for simplicity (a rectangular longitudinal section; a smooth curve; a completely eccentric lesion). Whilst this was necessary to demonstrate the haemodynamic relationships between anatomy and physiology, it is not directly applicable to real-world lesions. Further work is required in this area. Second, flow was modelled as steady and not pulsatile. However, previous work has established that this is unimportant in similar simulations with results being near identical to those with pulsatility.^27^ Third, only a limited number of permutations and combinations of the variables could be studied for reasons of time. Fourth, the MVR value was not personalized, and a population average was applied, although two limited variations (high and low MVR) were studied. Fifth, our absolute vFFR values, for the above reasons, may not reflect the exact value for an individual, but all the comparisons shown here (one value relative to another with a single parameter changed) are valid. Sixth, we did not study a lesion positioned exactly at a bifurcation, even though that is a common site, because the morphology is so variable. Lastly, when constructing branched geometries an angle of 45° was chosen purely for modelling simplicity because currently the workflow does not factor the angle of bifurcation into its calculations and instead relies on the assumptions mentioned in our methods above. However, this should not detract from the vFFR data generated because this is not meant to be a comprehensive study of branches. Our focus was purely on how varying the diameter of our daughter branches affects vFFR, through its impact on the distribution of MVR, and how this compares with straight vessels.

### Future direction

Ultimately, the work in this project may develop into a reduced order model (ROM) of CFD-derived vFFR. ROM development would extend the work of the current study, by increasing the number of variations, combinations and simulations by several thousand-fold to generate a more sophisticated and comprehensive ‘atlas’ of vFFR capable of interpolating and predicting vFFR instantaneously without the requirement for any case-specific CFD simulation. ROM models have demonstrated usefulness in a wide range of applications and are ideally suited to vFFR because they generate instant results apposite for clinical decision-making.

## Conclusion

We have used computational modelling to generate FFRs in a range of idealized geometries, intended to imitate commonly encountered patterns of coronary artery disease. By using computer modelling and idealized geometries, we were able to carefully control and vary commonly occurring anatomical features in order to investigate the strength of association between these features and their impact on FFR. Although invasive assessment with FFR or non-hyperaemic pressure ratios remains the gold standard for guiding coronary intervention these data help quantify the physiological effects of some specific simplified lesion and vessel combinations, challenge some assumptions and may add precision to regular visual angiographic assessment.

## Lead author biography

**Figure:**
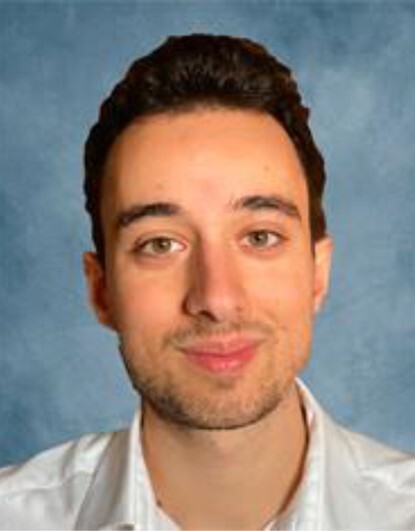


Roberto T.F. Newcombe is a 4th year medical student at the University of Sheffield, where he completed a BMedSci under supervising authors Professor Julian Gunn and Dr Paul Morris studying the effect of different lesion and vessel characteristics on virtual (v)FFR using the VIRTUheart™ workflow developed at the University of Sheffield.

## Supplementary material

Supplementary material is available at *European Heart Journal – Digital Health* online.

## Supplementary Material

ztab075_supplementary_data
