# Supplementary material for: The relationship between coronary stenosis morphology and fractional flow reserve: a computational fluid dynamics modelling study
Source: Eur Heart J Digit Health. 2021 Aug 15;2(4):616–25. doi: 10.1093/ehjdh/ztab075 (PMC9113079; doi:10.1093/ehjdh/ztab075)
Supplement: ztab075_supplementary_data [file ztab075_supplementary_data.docx]

**APPENDIX/SUPPLEMENTARY MATERIAL**

**Supplementary Table 1 : Examples of 40% and 90% concentric and eccentric lesions within a straight vessel with corresponding vFFR values**

|  | **Examples of geometries with 40% Diameter stenosis (DS)** | **Examples of geometries with 90% Diameter stenosis (DS)** |
| --- | --- | --- |
| **Concentric example** | 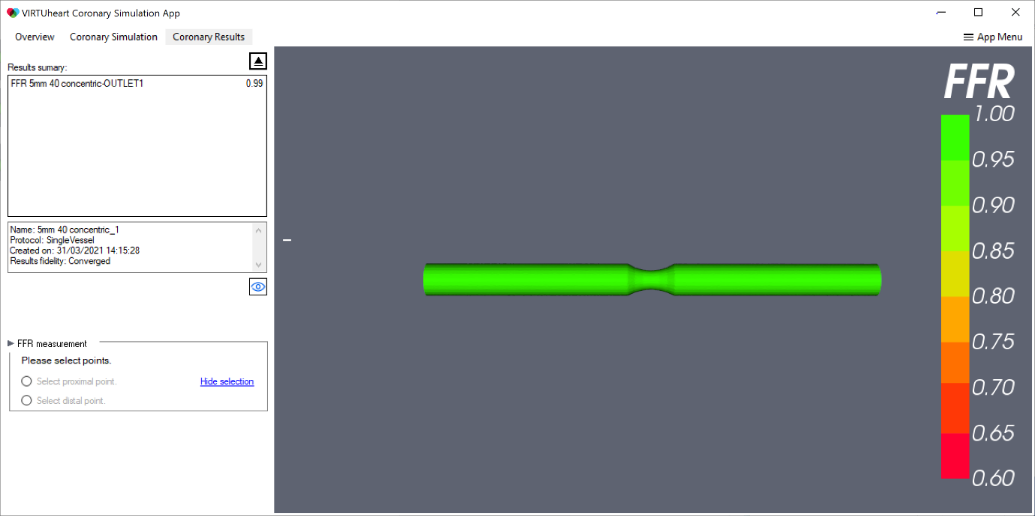  A vFFR – 0.99 | 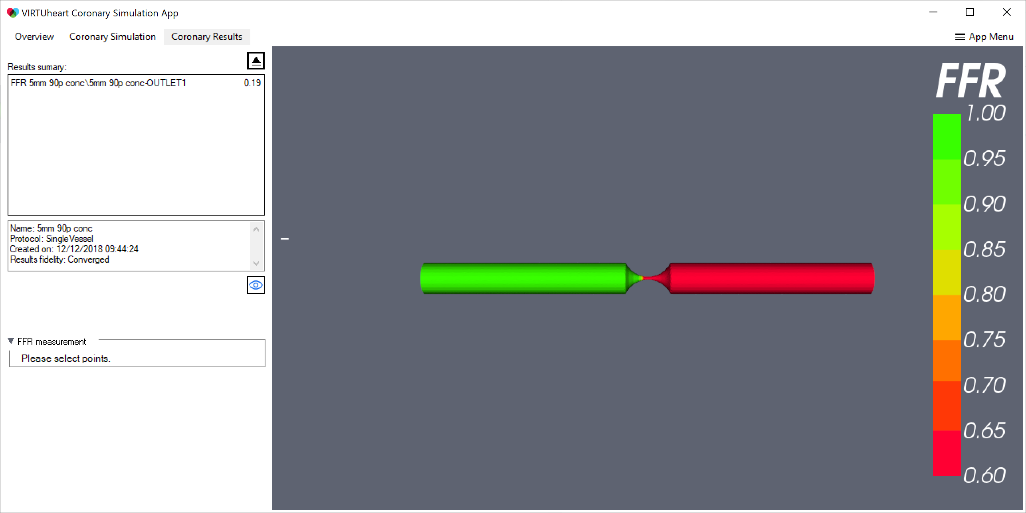  B vFFR – 0.19 |
| **Eccentric example** | 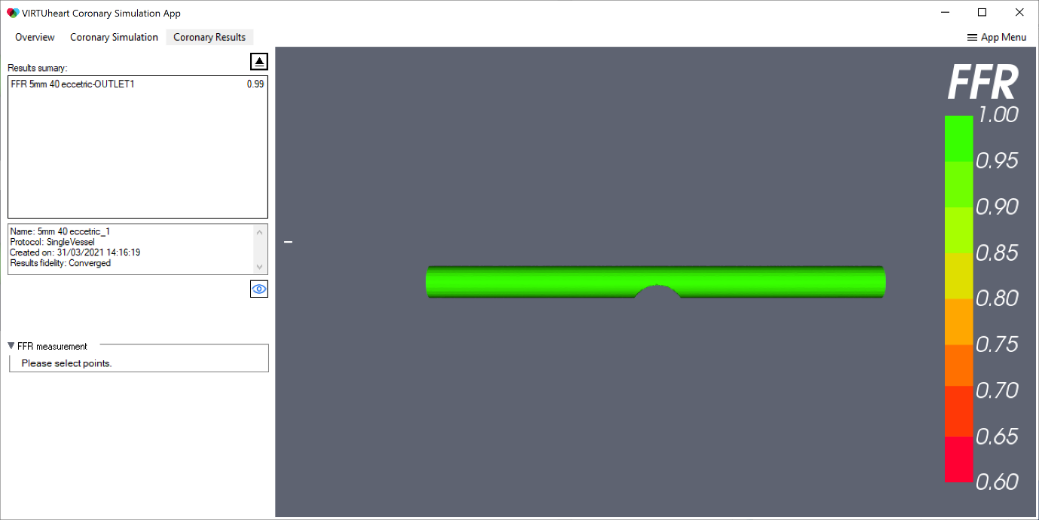  C vFFR – 0.99 | 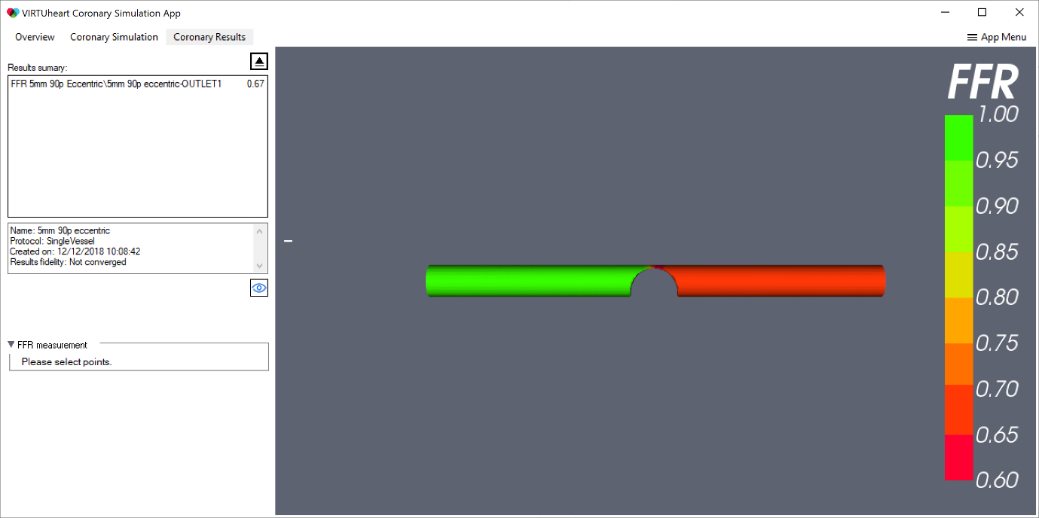  D vFFR – 0.67 |
| DS, diameter stenosis | | |

Appendix Table 1 legend: Examples of geometries containing lesions of either 40% or 90% DS. a.) A 40% concentric, rounded 5mm long focal stenosis. b.) A 90% concentric, rounded 5mm long focal stenosis. c.) A 40% eccentric, rounded 5mm long focal stenosis. d.) A 90% eccentric, rounded 5mm long focal stenosis
